# Impact of telemedicine adoption on accessibility and time to treatment in patients with thoracic malignancies during the COVID-19 pandemic

**DOI:** 10.1186/s12885-021-08819-z

**Published:** 2021-10-11

**Authors:** Vivek Nimgaonkar, Charu Aggarwal, Abigail T. Berman, Peter Gabriel, Lawrence N. Shulman, John Kucharczuk, Megan Roy, Joshua M. Bauml, Aditi P. Singh, Roger B. Cohen, Corey J. Langer, Melina E. Marmarelis

**Affiliations:** 1 Perelman School of Medicine, University of Pennsylvania, Philadelphia, PA USA; 2 Division of Hematology and Oncology, Department of Internal Medicine, Perelman School of Medicine, University of Pennsylvania, Philadelphia, PA USA; 3 Department of Radiation Oncology, Perelman School of Medicine, University of Pennsylvania, Philadelphia, PA USA; 4 Division of Thoracic Surgery, Hospital of the University of Pennsylvania, Philadelphia, PA USA

**Keywords:** Telemedicine, Time to treatment initiation, Accessibility, Thoracic malignancy

## Abstract

**Background:**

To ensure safe delivery of oncologic care during the COVID-19 pandemic, telemedicine has been rapidly adopted. However, little data exist on the impact of telemedicine on quality and accessibility of oncologic care. This study assessed whether conducting an office visit for thoracic oncology patients via telemedicine affected time to treatment initiation and accessibility.

**Methods:**

This was a retrospective cohort study of patients with thoracic malignancies seen by a multidisciplinary team during the first surge of COVID-19 cases in Philadelphia (March 1 to June 30, 2020). Patients with an index visit for a *new phase of care,* defined as a new diagnosis, local recurrence, or newly discovered metastatic disease, were included.

**Results:**

240 distinct patients with thoracic malignancies were seen: 132 patients (55.0%) were seen initially in-person vs 108 (45.0%) via telemedicine. The majority of visits were for a diagnosis of a new thoracic cancer (87.5%). Among newly diagnosed patients referred to the thoracic oncology team, the median time from referral to initial visit was significantly shorter amongst the patients seen via telemedicine vs. in-person (median 5.0 vs. 6.5 days, *p* < 0.001). Patients received surgery (32.5%), radiation (24.2%), or systemic therapy (30.4%). Time from initial visit to treatment initiation by modality did not differ by telemedicine vs in-person: surgery (22 vs 16 days, *p* = 0.47), radiation (27.5 vs 27.5 days, *p* = 0.86, systemic therapy (15 vs 13 days, *p* = 0.45).

**Conclusions:**

Rapid adoption of telemedicine allowed timely delivery of oncologic care during the initial surge of the COVID19 pandemic by a thoracic oncology multi-disciplinary clinic.

## Background

The COVID-19 pandemic has required the medical community to rethink the care delivery model for vulnerable populations such as patients with cancer. Patients with thoracic malignancies have an especially high risk of mortality following infection with the severe acute respiratory syndrome coronavirus 2 (SARS-CoV-2), potentially due to underlying structural lung disease and treatments used in this type of malignancy [1]. As a result, physicians have had to balance the competing risks of SARS-CoV-2 exposure through clinic visits and the danger of delayed treatment [2].

The timeliness of care delivery in oncology has been recognized as an important quality of care metric; delays in the delivery of care are associated with mortality in several malignancies [3]. For thoracic malignancies, guidelines have been established for the timely initiation of treatment following diagnosis [4–6]. Nevertheless, it has previously been observed that there can be considerable variation in time to treatment initiation between and within health systems [7, 8] with additional impacts on patient dissatisfaction and distress [9].

To address the unique competing risks imposed by the COVID-19 pandemic, our institution and others created guidelines for the management of thoracic malignancies during surges in COVID-19 infections [10–12]. Amongst the recommendations offered, adoption of telemedicine was identified as a method to reduce SARS-CoV-2 transmission. Telemedicine is defined by the use of information and communications technologies to provide medical care remotely. Though telemedicine can take many forms, this study focuses primarily on synchronous patient visits with a physician via a phone or video-conferencing service, which has been rapidly adopted across the United States in the care of patients with cancer, facilitated by policy changes that adjusted the reimbursement structure for telemedicine visits [13].

Limited data exist, however, on the direct impact of telemedicine on key quality of care metrics [14]. Preliminary studies during the pandemic have demonstrated that telemedicine utilization in the context of oncology practice can be rapidly adopted [15], though patient and provider perspectives on its utility vary [16–19]. Historically, telemedicine in oncology practice has focused on delivery of care to underserved rural areas [20–22], augmentation of global oncology efforts [23, 24], provision of palliative care [25], remote monitoring of symptoms and mental health [26], and facilitation of multidisciplinary coordination with radiology and pathology teams [27]. The widespread use of telemedicine during the initial visit with an oncologist is new, and the downstream implications on timeliness and quality of care have not been investigated.

Consequently, this study sought to evaluate the impact of telemedicine adoption on the timeliness of treatment initiation for thoracic malignancies during the COVID-19 pandemic at an academic medical center. This study focused on the question of whether care could be efficiently initiated if the initial visit with a provider occurred via telemedicine. In doing so, this study aims to provide preliminary evidence on the effects of telemedicine on quality of care metrics to inform further study and evaluation of telemedicine in the pandemic contexts and beyond.

## Methods

### Overview

This was a retrospective cohort study of patients with thoracic malignancies seen by a multidisciplinary team at the University of Pennsylvania Health System (UPHS) over four months encompassing the time period immediately preceding and including the first surge of COVID-19 cases in Philadelphia (March 1 to June 30, 2020). STROBE guidelines have been adhered to in the reporting of this observational study [28]. This study was granted IRB exemption for a quality improvement project by the University of Pennsylvania IRB.

### Participants

Patients with thoracic malignancies were identified via chart abstraction based on completing a visit with a surgical, medical, or radiation oncologist specializing in thoracic malignancies and a diagnosis of a lung, thymic, or pleural malignancy. Patients were excluded if they did not have a visit for a *new phase of care* during the specified time period. Visits for a *new phase of care* were defined as those for a new diagnosis, local recurrence, or newly discovered metastatic disease. Patients were excluded if they did not receive subsequent oncologic care within UPHS. Patients were divided into groups based on index visit type: in-person vs. telemedicine. It should be noted that the decision regarding index visit type was made at the time of visit scheduling by patients in consultation with a nurse navigator or other clinical staff.

### Measures

Dates of index visit and treatment initiation were abstracted from the electronic medical record (EMR) along with baseline demographic information including sex, gender, and diagnosis for all patients. Dates of referral were abstracted only for patients with new diagnoses who were new to the multidisciplinary thoracic oncology team as most patients with recurrent disease had already been seen by one of the physicians within the multidisciplinary team. Referral dates were identified through documentation by a nurse navigator, referring provider, or documentation from a hospital admission where a referral was made. For patients referred during a hospitalization, the date of discharge was used as the referral date. Referral dates were not always clearly recorded in the EMR and therefore could not be abstracted for all patients. For patients with a new diagnosis, time to index visit was defined as the days between the date of referral and the date of index visit. Time to treatment initiation was defined as the days from index visit to initiation of the recommended treatment.

### Statistical analysis

Baseline characteristics of the telemedicine and in-person groups were compared using the Fisher’s exact test and Wilcoxon-rank sum tests. Times to index visit were compared using the Wilcoxon-rank sum test. Comparisons of time to treatment initiation were made using the Wilcoxon-rank sum test. Non-parametric analysis tests were used in this study because normal distributions could not be assumed for the data; all statistical analysis was performed on Stata v14.4. Figures were created using R and Microsoft PowerPoint. All programs used for this study were chosen because of their availability to the investigators and convenience.

## Results

A total of 240 distinct patients with thoracic malignancies were seen for a new phase of care during the study interval. Among the 240 patients included in the study, 78 underwent surgery (32.5%), 58 were treated with radiation (including concurrent chemoradiation) (24.2%), and 73 received systemic therapy alone (30.4%). The majority of visits were for a new diagnosis of a thoracic cancer (87.5%). 132 patients (55.0%) were seen in-person and 108 (45.0%) were seen via telemedicine. Baseline characteristics of patients seen via telemedicine vs in-person were well balanced (Table 1). A higher proportion of initial visits were conducted via telemedicine when the patient received systemic therapy or radiation compared to surgery (Table 1). Additionally, a greater fraction of patients with recurrent disease were seen via telemedicine initially.

**Table 1:** Characteristics of patients by initial visit

	In-Person	Telemedicine	
	*N* = 132	*N* = 108	
Sex			
Female (%)	67 (50.8)	57 (52.8)	
Male (%)	65 (49.2)	51 (47.2)	
			*P* = 0.796
Age			
Median in Years (IQR)	69 (61, 75)	66 (60, 75)	
			*P* = 0.678
Race			
White (%)	96 (72.7)	79 (73.1)	
Black (%)	29 (22.0)	19 (17.6)	
Other/unknown (%)	3 (2.3)	7 (6.5)	
Asian (%)	4 (3.0)	3 (2.8)	
			*P* = 0.387
Diagnosis			
NSCLC (%)	101 (76.5)	75 (69.4)	
SCLC (%)	8 (6.1)	12 (11.1)	
Neuroendocrine (%)	10 (7.6)	6 (5.6)	
Mesothelioma (%)	5 (3.8)	6 (5.6)	
Thymoma (%)	4 (3.0)	2 (1.9)	
Other (%)	4 (3.0)	7 (6.5)	
			*P* = 0.438
Therapy Received			
Surgery (%)	65 (49.2)	13 (12.0)	
Radiation (%)	24 (18.2)	34 (31.5)	
Systemic therapy (%)	29 (22.0)	44 (40.7)	
No therapy received (%)	14 (10.6)	17 (15.7)	
			***P*** **< 0.001**
Phase of Care			
New diagnosis (%)	126 (95.5)	84 (77.8)	
Local recurrence (%)	4 (3.0)	8 (7.4)	
Metastatic recurrence (%)	2 (1.5)	16 (14.8)	
			***P*** **< 0.001**

**Fig. 1:**
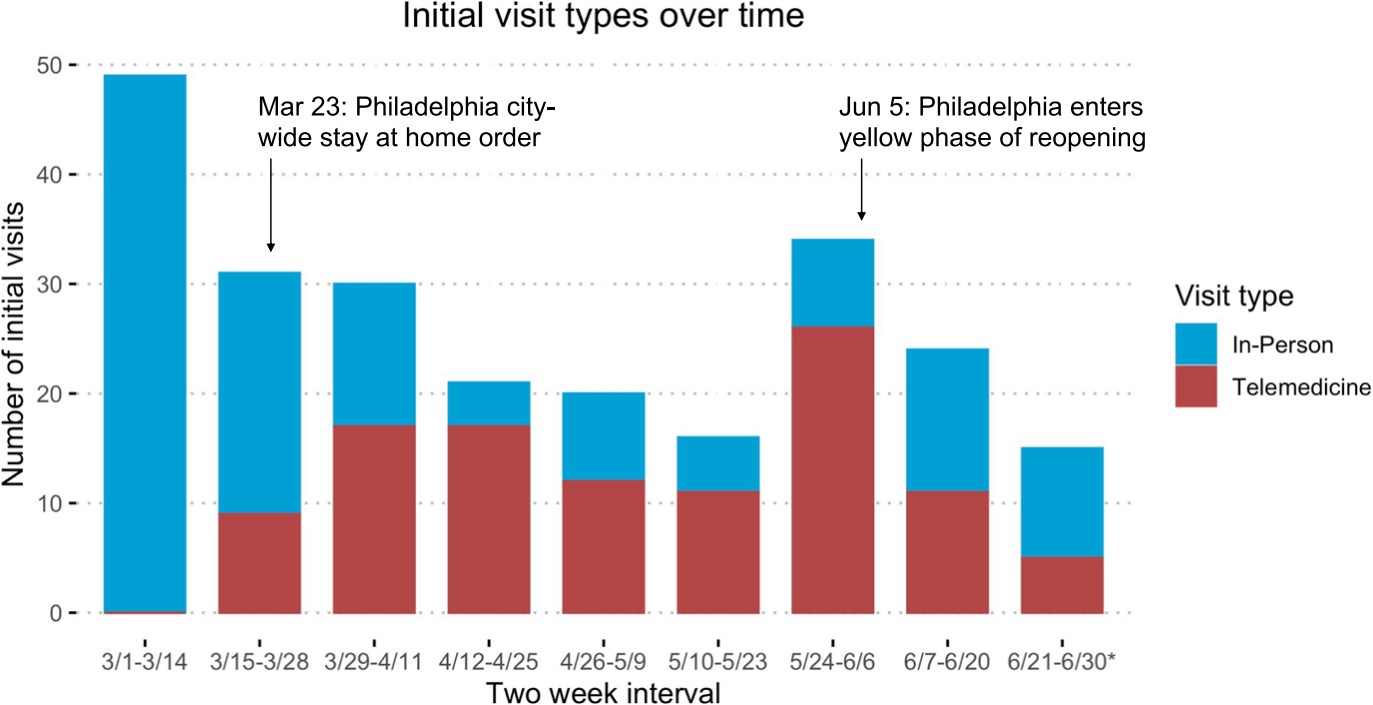
Telemedicine adoption during a COVID-19 surge. Number of telemedicine and in-person visits over two-week intervals between March 1 and June 30, 2020. 6/21–6/30, is 10, not 14 days

As expected, the proportion of telemedicine to in-person visit types changed with the local phase of the pandemic (Fig. 1). In the first two weeks of March prior to the initiation of a regional lockdown (Montgomery County- March 13, 2020, Philadelphia- March 23, 2020), none of the 49 patients seen for a new phase of care had an index visit conducted via telemedicine. An increased proportion of index visits were conducted via telemedicine in the ensuing weeks after a regional lockdown went into effect [29]. The proportion of in-person visits proceeded to rise in June 2020, during the re-opening phase.

The majority of patients received the recommended treatment: 78 of the 81 patients (96.3%) recommended for surgery, 73/77 (94.8%) recommended for systemic therapy, 58/63 (92.1%) recommended for radiation or chemoradiation. Patients not receiving recommended therapy, did not receive that treatment due to worsening performance status, decisions to pursue hospice care, death, insurance denying coverage of radiation, and patient preferences. For 19 patients, treatment was not recommended and not administered.

**Fig. 2:**
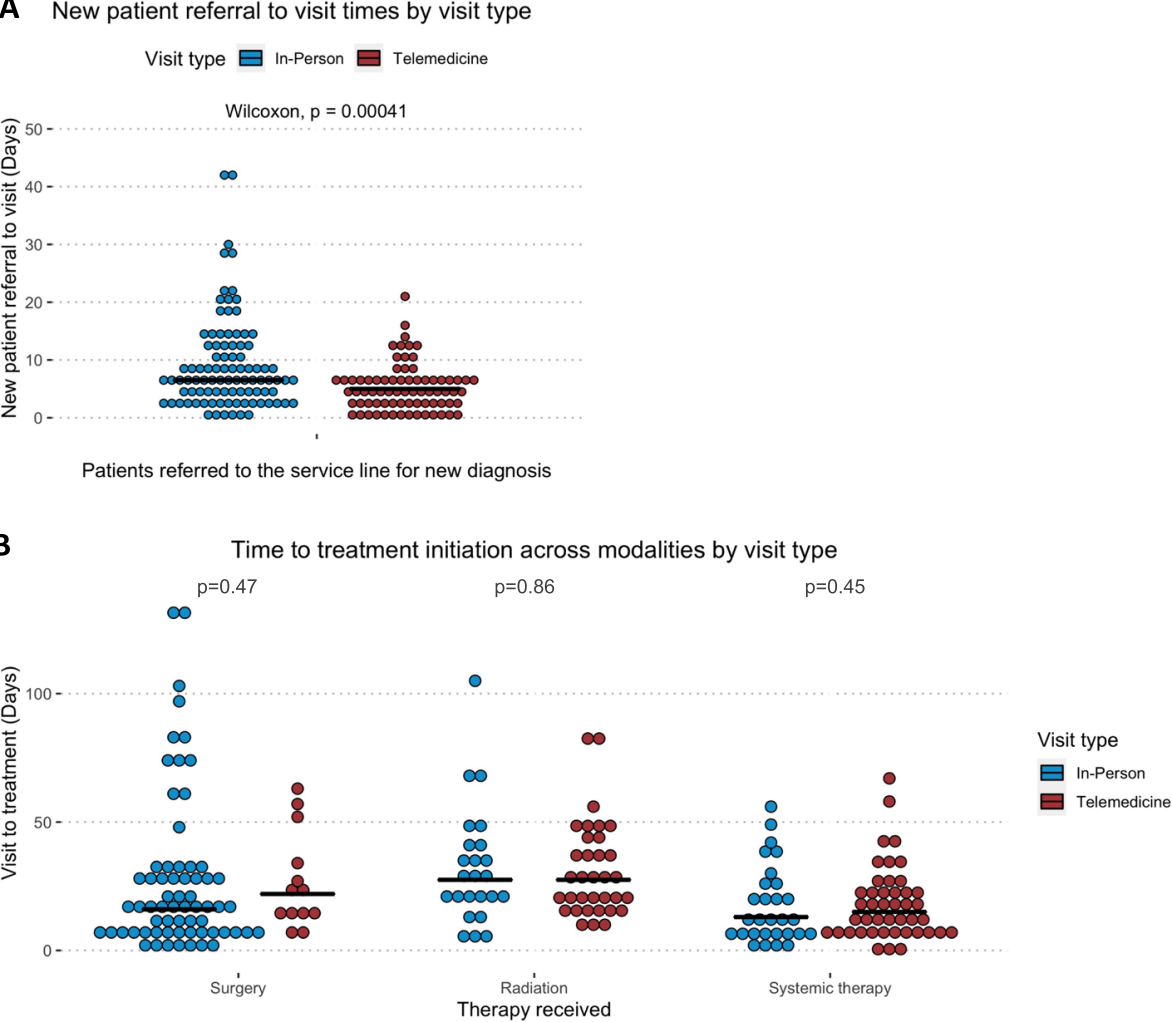
Time to oncologic care by visit type **A**. Time from the date of referral documented in the electronic health record to the date of initial visit for all patients with new diagnoses with median values labeled with bars (median values in-person vs. telemedicine: 6.5 vs. 5 days). Referral dates were available for 168 of 185 patients new to the service line referred for new diagnoses (90.8%); 16 in-person visits and 1 telemedicine visits were missing a referral date. **B**. Time from index visit to treatment initiation across different treatment modalities. Median values labeled with bars (median values in-person vs. telemedicine: Surgery 16 vs. 22 days; Radiation 27.5 vs. 27.5 days; Systemic therapy 13 vs. 15 days)

Of the 210 patients with a new diagnosis of a thoracic malignancy, 185 (88.1%) were new to the thoracic oncology multidisciplinary team. Among these 185 patients new to the team with a new diagnosis of a thoracic malignancy, the median time from referral to initial visit was shorter amongst the patients seen via telemedicine vs. in-person (5 vs. 6.5 days, *p* < 0.001) (Fig. 2A). Dates of referral could not be obtained from the EMR for 17 patients new to the service line (9.2%), 16 of which were in the in-person group and 1 of which was in the telemedicine group. Within the telemedicine group, it was noted that 2 patients were seen on the same day that a referral was received, and 12 patients were seen the day after referral. This compares to 1 same-day in-person visits and 5 in-person visits the day following referral.

Time to treatment stratified by treatment modality received did not differ by type of initial visit (in-person vs. telemedicine, Fig. 1C). Median time to treatment initiation for patients with in-person visits vs. telemedicine visits was 16 vs. 22 days for surgery (*p* = 0.47), 27.5 vs. 27.5 days for radiation (*p* = 0.86, and 13 vs. 14 days for systemic therapy (*p* = 0.45). A sensitivity analysis limited to new diagnoses only (210/241) confirmed the same results.

## Discussion

To date, studies of telemedicine in oncology have not addressed the impact of telemedicine utilization on the delivery of timely oncologic care, despite rapid adoption during the COVID-19 pandemic. This study builds on the work of other groups reporting the feasibility of rapid adoption of telemedicine in multi-disciplinary oncology settings and exploring its use in various other clinical contexts [30, 31]. Thus, it offers clinically relevant insights into the use of telemedicine in the care of oncology patients at a time when the ongoing COVID-19 pandemic continues to demand utilization of telemedicine.

This study provides preliminary evidence that index visits performed via telemedicine did not delay time to treatment in thoracic oncology patients. While the allocation of patients to a telemedicine or in-person index visit was not randomized, the populations of patients with telemedicine and in-person visits appeared broadly similar across age, gender, diagnosis, and race. Patients with recurrence, and likely an established relationship with a member of the multi-disciplinary team, were more likely to be seen via telemedicine for the index visit of their new phase of care. This could potentially introduce bias into the analysis favoring shorter time to treatment in the telemedicine group with a higher proportion of established patients. To address this concern, a sensitivity analysis of only new diagnoses was performed, demonstrating that the time to treatment did not differ between index visits conducted via telemedicine compared to in-person across the three treatment modalities.

Telemedicine is particularly challenging in patients considering surgery [32, 33]. In this study, a smaller proportion of surgical patients were seen initially via telemedicine, potentially reflecting physician or patient preference to meet in-person when considering surgery. However, this study did not find a difference in time to surgery when evaluating index visits conducted via telemedicine vs in-person, suggesting that preliminary surgical candidacy may be assessed via telemedicine for some patients. The limited number of telemedicine visits for surgical patients in this study highlights the need for further study of this patient population.

Given reports of significant delays in the receipt of treatment due to the COVID-19 pandemic, it was anticipated that delays to treatment delivery might be reflected across our study cohort. However, review of previous studies evaluating time to treatment initiation in thoracic malignancies prior to the pandemic suggests that time to treatment initiation in our cohort did not differ dramatically from the pre-pandemic standard of care. Direct comparisons are challenging because of heterogeneity in the definitions of time to treatment in the literature with time to treatment variably defined as the interval to treatment from pathologic diagnosis, first visit, or radiologic concern. However, time from initial visit with a specialist to treatment is used in several other studies. Vidaver et al. reported a median time to treatment initiation following specialist consultation of 27 days in a multi-site study of lung cancer patients outside of the pandemic setting [7]. This pre-pandemic estimate in a comparable population is consistent with findings during the pandemic in this study, suggesting that timely initiation of care was maintained during the pandemic possibly due to the utilization of telemedicine.

Comparisons to guidelines from expert bodies can be similarly challenging. Recommendations from the UK’s National Optimal Lung Cancer pathway call for treatment within 28 days of diagnosis; the Dutch national practice guideline for NSCLC states that treatment start within 35 days of a first pulmonology visit; the RAND corporation has set guidelines for treatment initiation within 42 days of NSCLC diagnosis and 14 days of SCLC diagnosis [4, 5, 7]. While it is beyond the scope of this study to address whether there were clinically meaningful delays attributable to the pandemic, the gross alignment of time to treatment initiation in our cohort with the pre-pandemic guidelines for timely initiation of care may provide preliminary evidence that telemedicine can help obtain and improve on these benchmarks outside of a pandemic setting. The generalizability of such findings has implications not just for clinicians and patients, but also for policy-makers weighing the reimbursement and regulatory framework for telemedicine in the absence of a pandemic or after the pandemic subsides.

A statistically significant difference in the time from referral to index visit was also observed in patients whose index visit was via telemedicine (5 days for telemedicine vs. 6.5 days for in-person). There are multiple potential mechanisms for this improvement with telemedicine, including the mitigation of traditional barriers to access such as transportation and limitations in clinic space. Though the clinical significance of this 1.5-day difference in the median time to initial visit is unclear, there may be meaningful differences in patient satisfaction and convenience enabled through an expedited time to initial visit. Previous assessments of the time from specialist referral to specialist visit for lung cancer patients have ranged from 1 to 17 days [8]. Further study will be required to address whether telemedicine can reduce patient perceptions of delays and improve satisfaction with care. Importantly, the analysis of time from referral to initial visit in this study was limited by the inability to report dates of referral for a share of patients (9.2% of patients referred to the service line). Prospective collection of these data in the future to ensure less missing data will help to better evaluate this question.

Several limitations of this study should be noted in weighing the conclusions. Missing data on referrals and an imbalance of new diagnoses and recurrent disease could introduce bias in our results as previously discussed. In addition, data on stage or burden of disease at diagnosis was not available, potentially representing a confounding variable that could affect times to treatment initiation. Finally, this study does not include data to address the outcomes of patients seen via telemedicine vs. those seen in-person, and consequently, it cannot assess the quality of the treatment provided beyond the time to initiation. Further study is needed to examine patient outcomes and additional quality metrics when receiving oncologic care via telemedicine.

There are several potential clinical implications of this study. The finding that treatment can be initiated in a similar time with the use of telemedicine visits suggests that telemedicine may effectively mitigate the delays in care during a pandemic when in-person visits are limited. The rapid incorporation of telemedicine into practice also supports the feasibility of quickly shifting to this mode of visit. Additionally, the reduced time from referral to initial visit seen in the telemedicine group in this study indicates that telemedicine visits offer a potential tool to expedite access to oncologic care.

In addition to the preliminary evidence offered related to accessibility and time to treatment initiation, this study supports the feasibility of rapid adoption of telemedicine to sustain the delivery of thoracic oncology care during surges of the COVID-19 pandemic and potentially in future pandemics. It should be noted that implementation of telemedicine requires resources (technological, financial, personnel) on both the provider and patient sides. The importance of these dimensions of telemedicine implementation should not be minimized and have been recognized by others [34]. Future studies should continue to focus on the necessary elements for efficient telemedicine delivery and the degree to which telemedicine exacerbates or ameliorates existing disparities in access to care.

## Conclusion

This study demonstrates that the time to treatment initiation did not differ following in-person and telemedicine visits in a multi-disciplinary thoracic oncology clinic after rapid adoption during the COVID-19 pandemic. Additionally, the time from referral to index visit was shorter for telemedicine visits than in-person visits in this study. Collectively, these findings suggest that the adoption of telemedicine into the care for thoracic oncology patients has the potential to facilitate the delivery of timely care and should be evaluated in further prospective studies that also interrogate additional quality of care metrics.

## Data Availability

We are unable to share the internal dataset used in this analysis with external parties.

## Abbreviations

COVID-19: Coronavirus Disease 2019
SARS-CoV-2: Severe Acute Respiratory Syndrome Coronavirus 2
UPHS: University of Pennsylvania Health System
STROBE: Strengthening the Reporting of Observational studies in Epidemiology
IRB: Institutional Review Board
EMR: Electronic Medical Record
